# Influence of wild-type *MLL *on glucocorticoid sensitivity and response to DNA-damage in pediatric acute lymphoblastic leukemia

**DOI:** 10.1186/1476-4598-9-284

**Published:** 2010-10-28

**Authors:** Alex H Beesley, Janelle L Rampellini, Misty-Lee Palmer, Jasmin YS Heng, Amy L Samuels, Martin J Firth, Jette Ford, Ursula R Kees

**Affiliations:** 1 Division of Children's Leukaemia and Cancer Research, Telethon Institute for Child Health Research, University of Western Australia Centre for Child Health Research, Perth, Australia; 2 Division of Biostatistics and Genetic Epidemiology, Telethon Institute for Child Health Research, University of Western Australia Centre for Child Health Research, Perth, Australia; 3 Curtin University of Technology School of Pharmacy, Perth, Western Australia

## Abstract

**Background:**

Rearrangement of the mixed-lineage leukemia gene (*MLL*) is found in 80% of infant acute lymphoblastic leukemia (ALL) and is associated with poor prognosis and resistance to glucocorticoids (GCs). We have recently observed that GC resistance in T-ALL cell lines is associated with a proliferative metabolism and reduced expression of *MLL*. In this study we have further explored the relationship between *MLL *status and GC sensitivity.

**Results:**

Negative correlation of *MLL *expression with GC resistance in 15 T-ALL cell lines was confirmed by quantitative RT-PCR. The absence of *MLL*-rearrangements suggested that this relationship represented expression of wild-type *MLL*. Analysis of *MLL *expression patterns revealed a negative relationship with cellular metabolism, proliferation and anti-apoptotic transcriptional networks. *In silico *analysis of published data demonstrated that reduced levels of *MLL *mRNA are associated with relapse and prednisolone resistance in T-ALL patients and adverse clinical outcome in children with *MLL*-rearranged ALL. RNAi knockdown of *MLL *expression in T-ALL cell lines significantly increased resistance to dexamethasone and gamma irradiation indicating an important role for wild-type *MLL *in the control of cellular apoptosis.

**Conclusions:**

The data suggests that reduced expression of wild-type *MLL *can contribute to GC resistance in ALL patients both with and without *MLL*-translocations.

## Background

Among pediatric subtypes of acute lymphoblastic leukemia (ALL), infants and those with T-lineage ALL are particularly resistant to glucocorticoids (GCs), one of the most important classes of drug for this disease [1]. Rearrangement of the mixed lineage leukemia gene (*MLL*) gene affects 80% of ALL in infants and is associated with a particularly poor prognosis [2,3]. *MLL *is located at 11q23 and encodes a histone methyltransferase that through its regulation of *HOX *genes is essential for normal mammalian development and hematopoiesis [4]. A unique feature of the *MLL *locus is that it is subject to an extremely wide variety of rearrangements, including translocations with >50 partner genes on various chromosomes, as well as deletions, inversions, internal duplications and gene amplifications [4-6]. There are conflicting reports on the relative GC responses of patients with different *MLL *translocations [7,8], but those with t(4;11) translocations appear particularly resistant [3,8,9]. The biological basis for the documented GC resistance of patients with *MLL*-disease has not been explored but has generally been assumed to be due to the oncogenic effects of translocated *MLL *fusion proteins.

Despite the clinical importance of GCs for the treatment of ALL, detailed knowledge about the transduction pathways leading to GC-induced apoptosis in lymphoid tissues remains limited [10]. Recently we performed transcriptional profiling of a panel of T-ALL cell lines and reported that GC resistance was associated with a proliferative metabolism [11]. We also observed that GC resistance profiles were significantly correlated with reduced expression of *MLL*. In this study we have further investigated the relationship between *MLL *expression and GC sensitivity in T-ALL and provide evidence that it is the wild-type expression of the gene, rather than the effect of translocations, that appears to be critical for determining a resistant phenotype. This novel finding may help to explain why GC-resistance is a common feature of most patients with *MLL*-disease despite the wide variety of possible gene rearrangements

## Methods

### Cell lines and drug sensitivity profiling

The cell line panel has been previously described and comprised nine T-ALL lines derived in our own laboratory from pediatric ALL bone marrow specimens (PER cell lines), plus six additional T-ALL cell lines obtained from external sources [12,13]. Cell lines were grown in RPMI-1640 supplemented with 2 mM L-glutamine, 10 nM 2-mercaptoethanol and 10-20% heat-inactivated fetal calf serum. The media for PER-cell lines contained additional non-essential amino acids and pyruvate, whilst 300 units/ml interleukin-2 is required for growth of PER-427 and PER-487. The sensitivity of the T-ALL cell lines to methylprednisolone (MPRED) and dexamethasone (DEX) has been previously published [12] and was measured using the MTT assay with drugs incubated over four days. The IC50 (drug concentration that inhibits cell growth by 50%) was used as the measure of drug resistance.

### Gene Expression Profiling

Briefly, RNA was extracted from cell lines in exponential growth phase and hybridized to Affymetrix HG-U133A microarrays [11,14]. Microarray data were normalized using robust multi-array analysis (RMA) and all passed quality control criteria for noise, background, absent/present calls, and 3'/5' signal ratios for *ACTB *and *GAPDH*. To interrogate the biological pathways represented by *MLL *expression profiles we used Gene Set Enrichment Analysis (GSEA) [15]. The median value of the five *MLL *probe sets present on the HG-U133A was calculated for each cell line, and correlated across the panel against all other probe sets on the array using Pearson's correlation as metric (GSEA v2.0, May 2006, 10,000 permutations). GSEA examines ranked lists of genes for enrichment of biological pathways contained within four different databases: C1 (genomic loci), C2 (curated biological pathways), C3 (genes with common regulatory motifs), and C4 (computational gene networks). Since not all genes within a given biological pathway are expected to be regulated in the same direction, rankings were performed using absolute correlation values as previously described [11]. Published microarray data used for *in silico *analysis [14,16-18] was downloaded from publicly available depositories or authors' websites.

### Real-time quantitative RT-PCR

Real-time quantitative RT-PCR (qRT-PCR) was performed on total RNA from cell lines in accordance with standard Applied Biosystems protocols (Foster City, CA) and in accordance with our published methods [19]. All experiments were run in duplicates on an ABI 7700 sequence detector and data normalized to expression of beta-actin (*ACTB*). Primers and probe for *MLL *and *GILZ *qRT-PCR were purchased from Applied Biosystems (ABI Assays on Demand); the *MLL *assay targeted exons 30-31 (Refseq NM_005933).

### RNAi knockdown of MLL expression

Three pSM2 retroviral RNAi vectors for *MLL *(V2HS_196843, V2HS_198375, V2HS_214961) and a non-silencing (NS) control vector were obtained from Open Biosystems (Huntsville, USA). For optimal mammalian expression, shRNA inserts were subcloned with EcoRI and XhoI into MSCV-LMP (MSCV/LTRmiR30-PIGΔRI, a generous gift from Prof. Scott Lowe, Cold Spring Harbour Laboratory [20]), which contains GFP and puromycin selection cassettes and drives miR30-shRNA expression using the retroviral 5'LTR. V2HS_198375 (MLL198) was found to suppress *MLL *expression most efficiently in transient transfection experiments and was used for subsequent experiments. The retroviral packaging cell line PA317 (selected in HAT medium) was transfected with linearised miR30-shRNA plasmid DNA (for both NS control and MLL198) using Lipofectamine, and GFP-positive cells were selected with puromycin. Stably transfected retroviral-producing PA317 cell lines were γ-irradiated (30 Gy) and incubated at 37°C overnight before co-culture with PER-117 cells for 48 hours. Retrovirally infected PER-117 cells were subsequently removed and selected with puromycin to generate cell lines stably expressing shRNA for *MLL *(MLL-KD) or the NS control (MLL-Scr). Efficiency of RNAi knockdown for *MLL *was assessed both by qRT-PCR as described above, and by immunoblot of nuclear protein extracted from cell lines in log-phase growth using standard methods. Antibodies used were mouse anti-MLL^C^/HRX, clone 9-12 (Upstate Cell Signaling Solutions, Temecula, CA), which detects the C-terminal proteolytic fragment of MLL (~180 kDa), and mouse anti-human β-actin as loading control (Pan Actin Ab-5 (ACTN05) NeoMarkers, Fremont CA). Densitometric quantitation of protein bands from multiple extractions taken at independent time points and from different cell-line stocks was performed using ImageJ software http://rsbweb.nih.gov/ij/, with MLL expression normalized to β-actin loading.

### Cellular assays

Cell growth and viability were measured using the Vi-CELL XR Viable Cell Analyzer (Beckman Coulter). Cells in exponential growth phase were seeded at 5 × 10^5 ^ml^-1 ^in a 96-well plate in the presence or absence of dexamethasone (10 μg/ml - 258 μg/ml, Mayne Pharma Pty Ltd, VIC, Australia), 0.025 μg/ml cytarabine (ARAC; Pharmacia Pty Ltd, NSW, Australia), 0.01 μg/ml methotrexate (MTX; David Bull Laboratories), or 1 Gy gamma-irradiation, and incubated for two days at 37°C before measuring cell survival. Each drug concentration or condition was tested in triplicate and data were normalised to values obtained from untreated cells. For metabolic assays, cells in exponential growth were seeded at 5 × 10^5 ^ml^-1 ^in fresh media and incubated for two days at 37°C before harvesting supernatants. Glucose and lactate supernatant concentrations were measured using the Amplex Red kit (Invitrogen, Australia), substituting lactate oxidase (Sigma, Australia) as required. For assessment of *GILZ *induction, MLL-KD and MLL-Scr cells in exponential growth were incubated with 1 μM dexamethasone (Mayne Pharma Pty Ltd, VIC, Australia) for four hours prior to RNA extraction and measurement by qRT-PCR.

## Results

### MLL mRNA Expression and GC resistance in T-ALL Cell Lines

**Figure 1:**
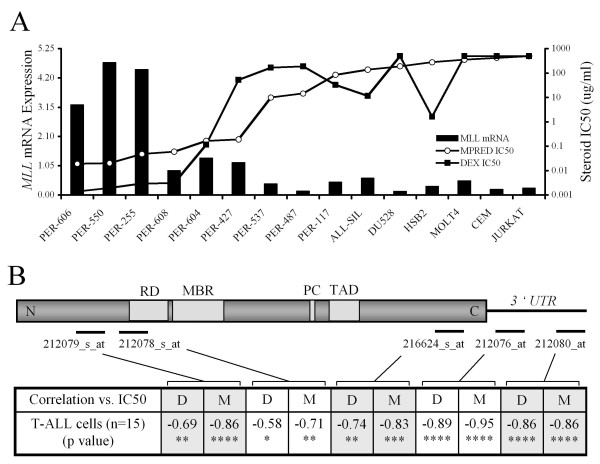
**Relationship between *MLL *expression and GC resistance in T-ALL Cell Lines**. (A) Normalized *MLL *mRNA expression across the T-ALL cell line panel as measured by qRT-PCR (bars) and IC50 values for MPRED (open circles) and DEX (closed squares); (B) Schematic of *MLL *mRNA indicating the target location of five microarray probes (indicated by solid lines) and protein domains within the coding region: MBR, Major Break Region; RD, Repression Domain; TAD, Transactivation Domain; PC, site of proteolytic cleavage. Correlation of expression level vs. cell line IC50 for DEX (D) and MPRED (M) is indicated for each probe (* p < 0.05, ** p < 0.01, *** p < 0.001, **** p < 0.0001).

Our laboratory has developed an authenticated panel of pediatric T-ALL cell lines that have been grown in the absence of drug selection. These cultures retain critical features of the primary disease and their drug resistance profile parallels the spectrum of resistance that has been observed in primary patient specimens [12]. We recently examined the baseline resistance of these 15 T-ALL cell lines to the GCs DEX and MPRED [12] and correlated the data with gene expression profiles as determined by HG-U133A microarray [11]. Although these lines have been cultured without prior exposure to *in vitro *drug selection pressure they demonstrate a natural spectrum of GC resistance, with IC50 values across the panel varying by 4-5 orders of magnitude (Figure 1A). This resistance profile is not explained by mutations in the glucocorticoid receptor (GR) or variations in its level of expression [21], indicating that defects downstream of the GR are primarily responsible for GC resistant phenotypes in these cell lines.

Our analysis of the microarray data revealed that GC resistance was significantly correlated with reduced expression of *MLL *[11]. To confirm this correlation we used qRT-PCR to measure *MLL *mRNA expression across the panel, using a probe targeting the 3' end of the *MLL *coding region. Expression levels measured by qRT-PCR were highly correlated with resistance to both GCs (Figure 1A; correlation vs. dexamethasone IC50 -0.849 (p < 0.0001), methylprednisolone IC50 -0.851 (p < 0.0001)). Whilst translocations of the *MLL *gene are prevalent in infant ALL they are infrequent in T-ALL [8,9,22], suggesting that the observed correlation reflected expression of the wild-type gene. Indeed, T-ALL cell line karyotypes indicated no abnormalities at the 11q23 *MLL*-locus [12], a conclusion confirmed by Southern Blot for all 15 cell lines (data not shown). On the HG-U133A microarray there are five independent probes for *MLL*, and these span the entire length of the gene, encompassing both sides of the major break region (MBR) that is involved in almost all translocation events (Figure 1B). Across the 15 T-ALL cell lines correlation of *MLL *mRNA expression and GC resistance was significant for all five probe sets (median probe significance DEX p = 0.0025, MPRED p < 0.0001) indicating no discrepancy in expression between the 5' and 3' regions of the gene. Based on these data we conclude that the observed correlation with GC sensitivity in T-ALL cell lines is related to expression levels of wild-type *MLL *rather than *MLL*-translocation products.

### Biological features of MLL expression in T-ALL

To gain further insight into the transcriptional programs associated with *MLL*, the expression profile of this gene across the T-ALL cell line panel was correlated to the expression of all other genes on the microarray. This output was analyzed with GSEA to identify the biological networks associated with variations in *MLL *expression. The strongest signatures were returned from the C2 (curated pathway) and C4 (computational gene network) databases, with 17 and 83 enriched gene sets respectively falling within the significant GSEA false discovery rate (FDR). Very few significantly enriched gene sets were identified from genomic loci and regulatory-motif databases (C1 and C3). The top ranked significant gene sets from the C2 and C4 databases are listed in Tables 1 &2. The majority of these pathways are involved with the control of cell growth and metabolism (e.g. *MYC*-regulated pathways, RNA transcription, oxidative phosphorylation, the TCA cycle, proteasomal regulation, nucleotide synthesis, translation initiation and antioxidant defense). The overwhelming direction of expression of these pathways was a negative correlation with expression of *MLL*. Thus lower expression of *MLL *in these cell lines is associated with signatures consistent with a proliferative phenotype. In addition the expression levels measured by each of the five *MLL *probe sets were found to correlate significantly with cell line doubling times [12] (median correlation 0.67, p < 0.01). These findings are in keeping with our previous observation that reduced expression of *MLL *is part of a proliferative metabolism signature that is associated with GC resistance in T-ALL cell lines [11]. Importantly, several gene sets were involved with the regulation of apoptosis (MORF_AATF, MORF_MAPK2), p53 response (MORF_EI24, GNF2_NS) and DNA damage repair (MORF_UNG), with the direction of association linking reduced *MLL*-expression with the activation of anti-apoptotic transcriptional networks (Table 2).

**Table 1:** Top ranked GSEA gene sets from the C2 database (curated pathways) associated with *MLL *expression profiles in T-ALL cell lines.

Gene Set	Description of Biological Pathway	FDR *
Electron_transport_chain	Electron transport chain	0.137

Glycolysis_gluconeogenesis	Glycolysis and gluconeogenesis	0.142

Peng_leucine_down	Down-regulated in response to leucine starvation	0.145

RNA_transcription_reactome	RNA transcription reactome	0.145

Mitochondria	Mitochondrial genes	0.148

Aminoacyl_tRNA_biosynthesis	Amino-acyl tRNA biosynthesis	0.151

Human_mitodb_6_2002	Mitochondrial genes	0.152

Hdaci_colon_cur24hrs_up	Genes upregulated by curcumin, transcription inhibitor	0.156

Hdaci_colon_cur48hrs_up	Genes upregulated by curcumin, transcription inhibitor	0.163

Myc_huvec_sage_array_up	Genes up-regulated by myc	0.176

Peng_rapamycin_down	Down-regulated in response to rapamycin	0.183

Oxidative_phosphorylation	Oxidative phosphorylation	0.190

tRNA_synthetases	tRNA synthetases	0.192

Proteasome_pathway	Proteasomal pathway genes	0.200

Peng_glutamine_down	Down-regulated in response to glutamine starvation	0.203

Krebs_TCA_cycle	Krebs (TCA) cycle genes	0.204

Proteasome	Proteasome genes	0.238

* FDR, false discovery rate; GSEA cutoff for significance FDR <0.25

**Table 2:** Top ranked GSEA gene sets from the C4 database (computed gene networks) associated with *MLL *expression profiles in T-ALL cell lines.

Gene Set	Description of Network Hub Genes and Associated Functions	FDR *
MORF_PRDX3	Peroxiredoxin 3 - MYC-mediated proliferation, glucose responses	0.132

MORF_SOD1	Superoxide dismutase 1 - mitochondria, oxidative metabolism	0.135

MORF_MAP2K2	MAP2K2 - ERK, JNK, p38, NFkB, and apoptosis pathways	0.138

MORF_PTPN11	Protein tyrosine phosphatase, cell growth, differentiation, metabolism	0.140

GNF2_RAN	RAS oncogene family - cell cycle, mitotic spindle regulation	0.143

MORF_GMPS	Guanine monphosphate synthetase - purine synthesis, cell cycle	0.146

MORF_DEAF1	DEAF1 or supressin, inhibitor of proliferation	0.152

MORF_ERH	Enhancer of rudimentary homolog - cell cycle regulator	0.155

GNF2_NS	Nucleostemin - cell cycle progression in stem cells, links with p53	0.158

MORF_GPX4	Glutathione peroxidase 4 - cellular antioxidant defence	0.159

MORF_AATF	Apoptosis antagonizing transcription factor	0.160

MORF_EIF3S2	EIF3S2 - eukaryotic translation initiation factor	0.164

MORF_ATOX1	ATX1 antioxidant protein 1 homolog - antioxidant defense	0.165

MORF_EI24	Etoposide induced mRNA - early p53 response gene	0.166

MORF_PSMC1	Proteasome 26S subunit, ATPase	0.173

MORF_RAN	RAS oncogene family - cell cycle, mitotic spindle regulation	0.179

MORF_RAB5A	Ras-associated protein - exocytosis, actin organisation	0.180

MORF_UNG	Uracil-DNA glycosylase - base-excision DNA repair pathway	0.182

MORF_FBL	Fibrillarin - component of snRNP synthesis of ribosomal RNA	0.203

GCM_MAX	Myc-associated factor X - transcriptional regulator	0.224

* FDR, false discovery rate; GSEA cutoff for significance FDR <0.25

### MLL-Translocation Partner Genes Correlate with MLL Expression

Recent evidence suggests that the genes most commonly translocated with *MLL *are not selected at random but may in fact be functionally related as part of an '*MLL*-web' [5,23,24]. If this is true, then in the context of the observed relationship between *MLL *expression and GC resistance in the present study (Figure 1A) one might predict that the expression of these genes would similarly be correlated with GC resistance in our T-ALL cell lines. Of the >50 known translocation partner genes of *MLL*, 43 are represented on the HG-U133A microarray (corresponding to a total of 93 probe sets). Despite the absence of *MLL*-translocations in the T-ALL cell lines we observed that a large number of these (18 genes, 26 probe sets) were significantly correlated to MPRED and DEX resistance (Table 3). This association is much greater than would be predicted by chance alone (exact binomial test, p < 0.001). It is relevant that the majority of the genes listed in Table 3 are involved in transcriptional regulation (*GMPS*, *DCPS*, *ELL*, *LPP*, *AF10*, *CREBBP*, *EP300*, *AF4*), proliferation (*GAS7*) or metabolism (*CBL*, *GPHN *and *ACACA*, the latter being the rate limiting enzyme for conversion of acetyl-coA into cholesterol). The correlation of these genes with GC resistance may therefore be reflective of the metabolic and proliferative changes driving this phenotype in T-ALL cell lines of which *MLL *appears to be a part [11].

**Table 3:** *MLL *Translocation Partner Genes Significantly Correlated with GC IC50 in T-ALL Cell Lines.

Probe Set	Gene Title	Symbol	Chr	MPRED	DEX*
220773_s_at	Gephyrin	*GPHN*	14q23.3	**0.915**	**0.670**
212186_at	Acetyl-CoA carboxylase alpha	*ACACA*	17q21	**0.767**	**0.558**
214431_at	Guanine monphosphate synthetase	*GMPS*	3q24	**0.754**	0.505
218774_at	Decapping enzyme, scavenger	*DCPS*	11q24.2	**0.637**	**0.744**
204096_s_at	Elongation factor RNA pol II	*ELL*	19p13.1	**0.581**	**0.779**
202821_s_at	LIM domain containing preferred translocation partner in lipoma	*LPP*	3q28	**0.577**	0.418
214358_at	Acetyl-CoA carboxylase alpha	*ACACA*	17q21	**0.569**	0.197
216506_x_at	*MLL *(trithorax homolog, Drosophila); translocated to, 10	*MLLT10/AF10*	10p12	**0.560**	0.293
200713_s_at	Microtubule-associated protein, RP/EB family, member 1	*MAPRE1*	20q11.1-11.23	**0.560**	**0.542**
211808_s_at	CREB binding protein (Rubinstein-Taybi syndrome)	*CREBBP*	16p13.3	**0.558**	0.470
215578_at	Gephyrin	*GPHN*	14q23.3	**0.526**	0.473
209768_s_at	SEPT5	*SEPT5/PNUTL*	22q11.21	**0.519**	0.229
216503_s_at	*MLL *(trithorax homolog, Drosophila); translocated to, 10	*MLLT10/AF10*	10p12	**0.516**	0.307

211067_s_at	Growth arrest-specific 7	*GAS7*	17p13.1	-0.300	**-0.541**
210872_x_at	Growth arrest-specific 7	*GAS7*	17p13.1	-0.334	**-0.580**
202191_s_at	Growth arrest-specific 7	*GAS7*	17p13.1	-0.385	**-0.624**
202192_s_at	Growth arrest-specific 7	*GAS7*	17p13.1	-0.417	**-0.589**
202221_s_at	E1A binding protein p300	*EP300*	22q13.2	**-0.542**	-0.315
212288_at	Formin binding protein 1	*FNBP1*	9q34	**-0.575**	**-0.624**
209027_s_at	Abl-interactor 1	*ABI1*	10p11.2	**-0.580**	-0.417
205068_s_at	Rho GTPase activating protein 26	*ARHGAP26*	5q31	**-0.607**	**-0.661**
209028_s_at	Abl-interactor 1	*ABI1*	10p11.2	**-0.608**	-0.472
214298_x_at	Septin 6	*SEPT6*	Xq24	**-0.615**	-0.386
201924_at	MLLT2	*MLLT2/AF4*	4q21	**-0.660**	**-0.671**
206607_at	Cas-Br-M (murine) ecotropic retroviral transforming sequence	*CBL*	11q23.3	**-0.681**	-0.456
213579_s_at	E1A binding protein p300	*EP300*	22q13.2	**-0.742**	-0.404

* Correlation coefficient (r) of probe set expression with MPRED or DEX IC50 values; Chr, chromosomal locus; bold values, p < 0.05. Genes are grouped together by positive (top) or negative correlation (bottom).

### Reduced MLL Expression in T-ALL Patients is Associated with GC Resistance and Relapse

**Figure 2:**
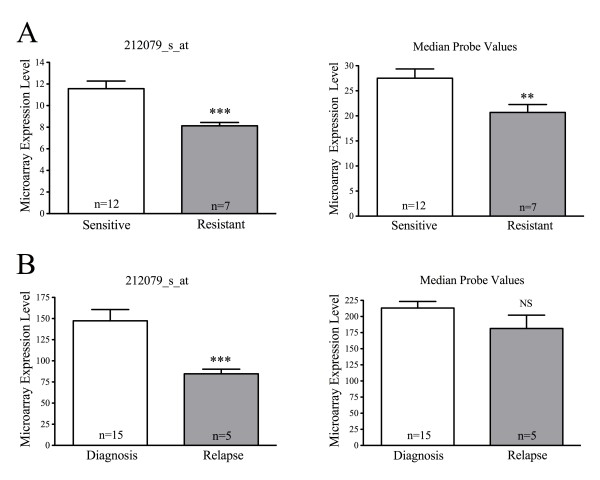
**Reduced *MLL *expression is associated with GC resistance and relapse in T-ALL patients**. Published HG-U133A microarray datasets were examined for the expression of *MLL *using either a single probe (212079_s_at) or the median of all five *MLL *probe sets on the array. Data represent the mean ± SEM (linear scale) of the indicated patient numbers (n). (A) Difference in *MLL *expression in pediatric T-ALL patient specimens with *ex vivo *sensitivity or resistance to prednisolone [17]; (B) Difference in *MLL *expression in pediatric T-ALL patient specimens measured at diagnosis or relapse [14].

Since our data indicated an association between GC sensitivity and expression levels of *MLL *in T-ALL *in vitro *we looked for further evidence in the literature for such an association. Holleman *et al *previously examined the *ex vivo *sensitivity of diagnostic pediatric ALL patient specimens to individual induction therapy agents and correlated the findings with gene expression data measured in the same samples using HG-U133A Affymetrix microarrays [17]. We examined this data for the expression level of *MLL *in T-ALL patient specimens from this cohort that were determined to be sensitive or resistant to prednisolone. Importantly, three of the five *MLL *probe sets on the array showed a significantly lower expression of *MLL *in resistant samples confirming the association we observed in T-ALL cell lines. Figure 2A shows the data for the probe set with the strongest association (212079_s_at, p < 0.001 unpaired t-test), and for the summary of the five probe sets calculated using median expression values (p < 0.05, unpaired t-test). For further evidence of a link between *MLL *expression and GC resistance we examined a dataset we have previously published comparing gene expression patterns in pediatric ALL specimens taken at the time of diagnosis and relapse [14]. Although we were not able to directly measure the GC sensitivity of these specimens it is known that almost all patients initially respond to induction therapy and achieve first remission, whilst GC resistance is a well-documented feature of relapse [25,26]. It is therefore reasonable to expect that many of the relapse specimens in this cohort would have elevated GC resistance compared to their diagnostic counterparts. Examining the same *MLL *probe sets as above, we observed a decrease in *MLL *expression in T-ALL relapse specimens vs. diagnosis specimens (Figure 2B) comparable to that measurable in GC resistant vs. sensitive specimens [17] (Figure 2A). This differential was only statistically significant for probe set 212079_s_at (p < 0.001, unpaired t-test), but the same trend was visible for the other four probe sets and is reflected in the summary of the median expression values for all five probes (Figure 2B). Since both of these studies involve T-ALL patients it is likely that the majority of patients within these cohorts do not have rearrangements affecting *MLL*. Taken together, this data provides clear support from two independent data sets that the correlation we have observed between wild-type *MLL *expression and GC sensitivity in T-ALL *in vitro *appears to also be relevant *in vivo*.

### Relevance of MLL Expression Level in Patients with MLL-Disease

**Figure 3:**
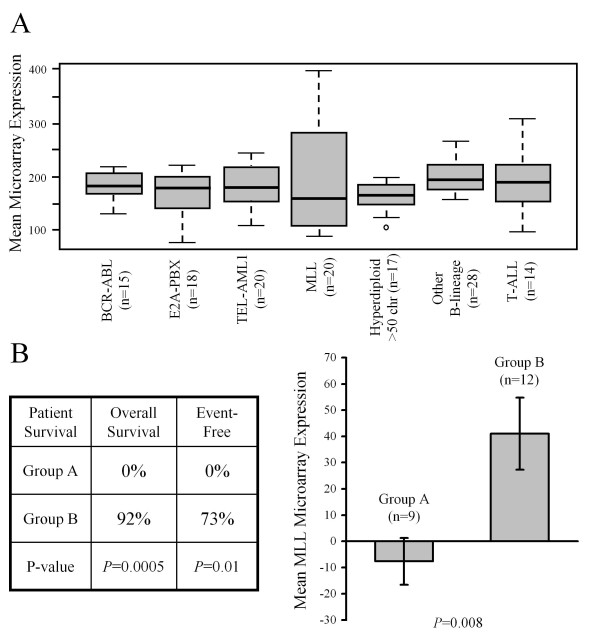
***MLL *expression patterns in patients with *MLL*-disease**. (A) Box and whisker plot of published microarray data [18] showing the variation of *MLL *expression in different ALL subgroups (HG-U133Plus2 probe 212076_at); (B) Analysis of published microarray data from ALL patients with *MLL*-rearrangements [16]. This study described two clusters of patients (A and B) with significantly different survival rates (boxed data) and expression of *MLL *(graph, mean ± SEM).

In our T-ALL cell lines we observed a 35-fold variation in *MLL *expression across the panel that correlated with GC resistance (Figure 1A). To assess the degree with which endogenous *MLL *expression levels vary in primary ALL patient specimens we analyzed data published by Ross *et al *who performed gene expression profiling of pediatric ALL subtypes [18]. Figure 3A shows that of all the pediatric ALL subtypes, the widest variations in *MLL *expression levels are found in patients with T-lineage ALL and those with *MLL*-rearrangements. To examine the prognostic relevance of *MLL *expression variation in patients with *MLL*-disease we examined a publication describing the use of Affymetrix HG-U95v2 microarrays to examine gene expression patterns in ALL patients with *MLL*-rearrangements [16]. These authors reported that such patients could be clustered on the basis of their genome-wide transcriptional profile into two distinct subgroups (called A and B) that demonstrated dramatically different survival rates (Figure 3B, box, p = 0.0005). By analyzing the data from their study we have ascertained that the expression of *MLL *was significantly lower in poor-outcome patients (Group A) compared to those with good outcome (Figure 3B, bar chart, p = 0.008). The HG-U95v2 probe for *MLL *targets the 3' UTR of the gene, meaning that it would either detect expression of the full-length (non-translocated) *MLL *allele remaining in these patients, or the expression of any reciprocal fusion that was transcribed as far as this 3' probe. Certainly it would not detect signal from primary *MLL*-translocation products. While the authors did not experimentally determine GC sensitivity in their study [16], the data are consistent with the hypothesis that the level of wild-type *MLL *expression is linked to therapeutic outcome even in patients that have an *MLL*-translocation on the alternate allele.

### MLL Knockdown Increases Resistance to GC Exposure and DNA Damage

**Figure 4:**
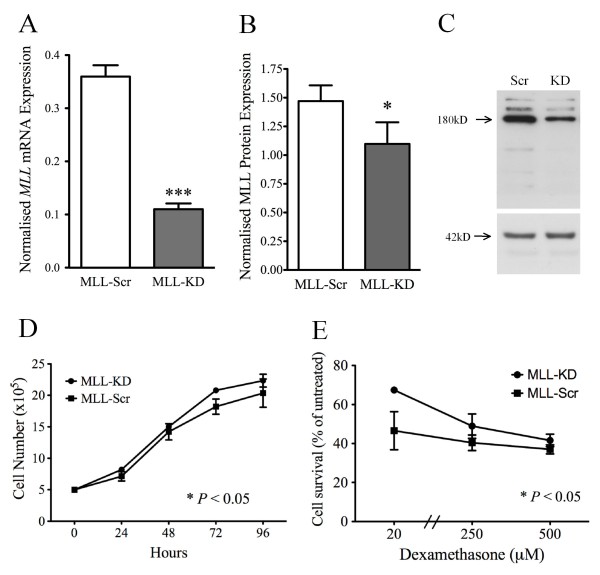
**Effect of *MLL *knockdown on proliferation and sensitivity to dexamethasone**. (A) Level of stable *MLL *mRNA knockdown in MLL-KD cells as measured by qRT-PCR compared to MLL-Scr cells expressing non-silencing scrambled shRNA; (B) Reduction of MLL nuclear protein expression in MLL-KD cells as assessed by immunoblot detection of the MLL^C ^proteolytic fragment from four independent extractions and normalized to β-actin loading control; (C) Representative immunoblot of nuclear MLL^C ^(~180 kD, top panel) and β-actin (42 kD, bottom panel) protein expression in MLL-Scr and MLL-KD cell lines; (D) Proliferation of MLL-Scr and MLL-KD cell lines over four days; (E) Differential growth of MLL-Scr and MLL-KD cells over two days in the presence of dexamethasone; In each case (A-B, D-E) data represent mean ± SEM from 3-6 independent experiments; Statistical analysis was by t-test (A, B) or ANOVA (D, E); * p < 0.05, *** p < 0.001.

**Figure 5:**
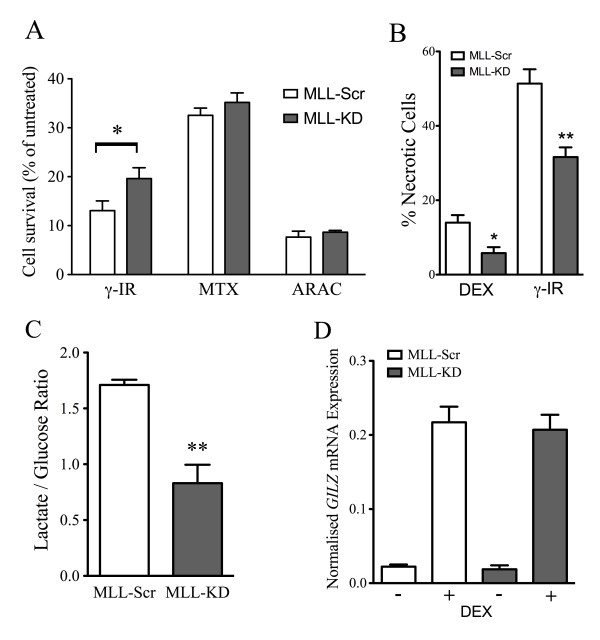
**Effect of *MLL *knockdown on cellular resistance, metabolism and GC signaling**. (A) Effect of gamma-irradiation (γ-IR, 1 Gy), methotrexate (MTX, 0.01 μg/ml), cytarabine (ARAC, 0.025 μg/ml) on growth of MLL-Scr and MLL-KD cell lines over two days; (B) Percentage of dying or necrotic cells (normalized to untreated) in MLL-Scr and MLL-KD after 48 hours in the presence of dexamethasone (DEX, 250 μM or 500 μM) or following gamma-irradiation (γ-IR, 1 Gy); (C) Ratio of lactate production/glucose consumption over two days in MLL-Scr and MLL-KD cells; (D) Relative expression of *GILZ *mRNA as measured by qRT-PCR in MLL-Scr and MLL-KD cell lines incubated for four hours in the absence (-) or presence (+) of dexamethasone (1 μM); In each case (A-D) data represent mean ± SEM from 3-6 independent experiments with statistical analysis by unpaired t-test; * p < 0.05, ** p < 0.01.

To assess the role of wild-type *MLL *in GC resistance phenotypes we used a retroviral RNAi expression system in the PER-117 T-ALL cell line to generate cell lines stably expressing shRNA for *MLL *(MLL-KD) or a non-silencing shRNA scrambled control (MLL-Scr). *MLL *mRNA expression in MLL-KD cells was 69% lower on average than in MLL-Scr control cells as assessed by qRT-PCR (Figure 4A, p < 0.0001). This correlated to a ~20% reduction in MLL nuclear protein as assessed by immunoblot (Figures 4B and 4C, p < 0.05). Proliferation assays demonstrated that MLL-KD cells grew approximately 10% faster on average than MLL-Scr cells (Figure 4D, p < 0.05 ANOVA). To assess GC sensitivity in the two lines, cell viability was assessed after a two-day incubation with dexamethasone (Figure 4E). MLL-KD demonstrated increased viability compared to MLL-Scr cells at all doses tested (p < 0.05, two-way ANOVA) indicating GC resistance. To assess the specificity of this protective effect we examined the sensitivity of the cells to gamma-irradiation, and incubation with cytarabine (ARAC) and methotrexate (MTX). Interestingly, MLL-KD cells showed greater survival following gamma-irradiation indicating resistance to DNA damage (Figure 5A, p < 0.05 unpaired t-test). Resistance to ARAC and MTX however was not significantly different between the two cell lines. The proportion of dying (necrotic) cells after two days was significantly reduced in MLL-KD cells in response to both dexamethasone and gamma-irradiation, indicating a cytoprotective effect of *MLL *knockdown (Figure 5B). Baseline viability in untreated cells was not significantly different between the cell lines.

To assess the effects of *MLL *knockdown on cell metabolism we compared rates of glucose consumption and lactate production between the two cell lines. Consistent with an increased rate of proliferation MLL-KD cells demonstrated an increased rate of glucose consumption compared to control cells. This was accompanied by a decreased rate of lactate production, resulting in a significant drop in the lactate production:glucose consumption ratio in MLL-KD cells (Figure 5C). Finally, since *MLL *is known to be a master transcriptional regulator we assessed whether the GC resistant phenotype of MLL-KD cells might represent transcriptional suppression of GC response elements by measuring the induction of *GILZ*, a well-characterized GC-response gene, following incubation with dexamethasone. There was no significant difference in the induction of *GILZ *mRNA between MLL-KD and MLL-Scr cell lines following a 4 hour incubation with dexamethasone (Figure 5D), indicating that GC-transcriptional responses in MLL-KD cells appeared to be intact.

## Discussion

Although there are conflicting reports of the effect of *MLL*-rearrangements on steroid resistance [7,8], it is clear that all infants with *MLL*-rearrangements have significantly worse prognosis than those with non-rearranged *MLL *regardless of the type of translocation involved [3,27]. However, the present study has been conducted using T-ALL cell lines without *MLL*-translocations and provides evidence that in the absence of such translocations cellular GC sensitivity is related to the level of expression of wild-type *MLL*. One interpretation of this data is that alterations in *MLL *support the proliferative phenotype that we have previously associated with GC resistance [11]. In lymphocytes, GCs are thought to trigger a metabolic crisis that ultimately leads to apoptosis [28]. In addition to suppressing apoptotic potential through the modulation of mitochondrial energetics, up-regulation of biosynthetic and metabolic pathways to support proliferation may therefore confer GC resistance by offsetting the adverse metabolic consequences of GC signalling [11]. *MLL *has recently been shown to be important for the control of cell proliferation but the mechanism is complex, involving a bimodal pattern of expression throughout the cell cycle [29]. In our experiments, suppression of *MLL *was associated with a small increase in proliferation and glucose consumption but decreased lactate production, indicating a shift away from aerobic glycolysis to alternative pathways, such as oxidative phosphorylation or the pentose-phosphate shunt. Besides energy production, these pathways are essential for the synthesis of macromolecules, nucleotides and nucleic acids required for proliferation [30].

In addition to elevated GC resistance, knockdown of *MLL *expression was associated with increased resistance to gamma-irradiation indicating an unexpected protection from the effects of DNA-damage. Recently it has been demonstrated that the *MLL *family of H3K4 methyltransferases are critical components of an E2F1-signalling pathway that mediates links cell cycle control to DNA damage responses, and that their knockdown attenuates the apoptotic response to adriamycin [31]. This highlights the tumor suppressor role of these proteins and is consistent with the protection from DNA-damage we have observed following *MLL*-knockdown in T-ALL cell lines. In contrast however, no protective effect of *MLL*-knockdown was seen for ARAC or MTX in the present study. Whilst one might expect that suppression of DNA-damage response pathways should increase resistance to both of these agents, it is interesting to note that, unlike GCs, elevated resistance to neither of these drugs is associated with *MLL*-rearrangement [32,33]; infants in fact are known to be generally more sensitive to ARAC [8,32]. There may therefore be some unexplained insult specificity in the role of *MLL *in mediating responses to DNA-damage.

Across the T-ALL cell lines there was a 35-fold variation in the level of *MLL*-expression. Surprisingly the mechanisms controlling expression of wild-type *MLL *have not been extensively studied, with most work focusing on the downstream effects of the gene and its various fusion products. However the putative *MLL *promoter has binding motifs for a large number of transcription factors, including *SREBF1 *(sterol regulatory element binding transcription factor) and *MYC*. *MYC *is a pivotal player in the control of cell cycle and apoptosis [34], is one of the known downstream targets of GC signaling in lymphocytes [35], and has been reported to be up-regulated in *MLL*-disease [36]. *MLL *expression is also likely to be subject to miRNA control, with numerous miRNA binding sites predicted to reside in the *MLL *3'UTR. Although downstream effects of *MLL *or *MLL*-translocations on miRNA expression has been reported by a number of groups, to our knowledge only one recent study has reported the upstream miRNA regulation of *MLL *itself [37]. In that study ectopic expression of mirR-221 and miR128 was shown to affect levels of *MLL*, *MLL*-fusions and GC sensitivity in ALL cell lines [37], consistent with the hypothesis that levels of *MLL *expression are important for GC resistance. It remains to be seen whether the observed effects of miRNA ectopic expression on GC sensitivity were due to effects on *MLL*-fusion proteins or endogenous wild-type *MLL *and the hierarchy for these mechanisms therefore remains to be untangled.

How do the present findings, performed in T-ALL with no *MLL*-translocations, relate to patients with *MLL*-disease? Although loss-of heterozygosity (LOH) at the *MLL *locus has been reported to be a relatively frequent event in childhood ALL, consistent with a potential role as a tumor suppressor [38], this is not the case in patients with *MLL*-disease where one wild-type copy of *MLL *appears to be retained [38-40]. This indicates that allele loss and *MLL*-translocation are mutually exclusive oncogenic events, but little focus has been given to the regulation of the remaining wild-type allele following translocation. However Whitman *et al *have recently demonstrated that in myeloid leukemia *MLL *partial tandem duplications (PTD) are associated with silencing of the wild-type *MLL *copy through an autoregulatory mechanism involving altered methylation [41]. Interestingly, in one MLL-PTD patient wild-type *MLL *was expressed at diagnosis but absent at relapse, suggesting a correlation with disease progression. Wild-type *MLL *expression could be re-induced in primary blasts with the use of DNA methyltransferase (DNMT) or histone deacetylase (HDAC) inhibitors, or suppression of the MLL-PTD transcript, and was associated with increased apoptotic sensitivity and reduced colony-forming capability. Other workers have recently demonstrated down-regulation of wild-type *MLL *in myeloid leukemia patients with different types of rearranged-*MLL *[42] suggesting that it may be a common feature of *MLL*-related leukemia.

## Conclusions

Based on the evidence presented we hypothesize that GC resistance in patients with *MLL*-disease may partly result from decreased expression and tumor suppressive effects of wild-type *MLL*, either through a gene-dosage effect following the functional loss of one allele via translocation, auto-regulation from the *MLL*-fusion protein, or altered miRNA/transcription factor signaling. This would help to explain why GC-resistance is a common feature of most patients with *MLL*-disease despite the wide variety of possible gene rearrangements. Amplifications of the *MLL *gene do occur but are much more rare. To our knowledge only one report exists where such a patient has been tested for *ex vivo *GC sensitivity [43] - in that small study a single patient with *MLL *amplification demonstrated GC sensitivity whilst all patients with *MLL *deletions or rearrangements demonstrated GC resistance, observations entirely consistent with our hypothesis.

We do not propose that the *MLL*-translocation event itself is without oncogenic effects since this has been clearly demonstrated by other workers, but rather that our data may help to explain the poor-response to therapy in this disease. Neither do our findings negate the possibility that *MLL*-fusion proteins themselves may have additional effects upon apoptotic sensitivity. Indeed, recent experiments have shown that multiple *MLL*-fusion proteins inhibit p53 and confer resistance to DNA damage [44]. However, it is important to note that in these experiments fusion protein constructs were ectopically expressed into cell lines containing wild-type *MLL*. In view of the evidence discussed here it would be important to know whether expression of endogenous *MLL *was altered during these experiments and whether this contributed to the observed anti-apoptotic effects. Increased resistance to DNA damage-induced apoptosis has been proposed as a phenotype of *MLL*-disease that explains the short latency associated with disease emergence [45]. It is possible that this effect could originate from the loss of tumor suppressor function of the wild-type *MLL *as well as from direct anti-apoptotic effects of the fusion protein.

During the preparation of this manuscript Liu *et al *[46] published a report describing a role for wild-type MLL in the maintenance of genome integrity through the regulation of the S-phase cell cycle checkpoint. DNA synthesis in cells deficient in wild-type MLL was found to be resistant to ionizing radiation and a range of DNA-damaging agents, supporting a role for wild-type MLL in the mediation of cellular DNA damage responses [46]. Under this model, MLL-fusion proteins acted as dominant negative mutants to abrogate the ATR-mediated stabilization of wild-type MLL reported to occur in response to DNA damage. The findings are in keeping with those from the present study and support our conclusion that reduced levels of wild-type MLL can contribute to increased cellular resistance even in the absence of an MLL-translocation event.

## Conflicts of interests

The authors declare that they have no competing interests.

## Authors' contributions

AHB directed research, analyzed data, prepared manuscript; JLR, MLP, JYSH, ALS, JF performed research, collected and analyzed data; MJF performed bioinformatics and statistical analysis; URK designed study, directed research, revised manuscript. All authors read and approved the final manuscript.
